# Pharmacological Inhibition of Brain Fatty Acid Binding Protein Reduces Ethanol Consumption in Mice

**Published:** 2017-10-31

**Authors:** Antonio Figueiredo, John Hamilton, Matthew Marion, Kenneth Blum, Martin Kaczocha, Samir Haj-Dahmane, Dale Deutsch, Panayotis K. Thanos

**Affiliations:** 1 Behavioral Neuropharmacology and Neuroimaging Laboratory on Addictions, Research Institute on Addictions, University at Buffalo, Buffalo, NY, USA; 2 Department of Psychiatry and McKnight Brain Institute, University of Florida College of Medicine, Gainesville, FL, USA; 3 Department of Anesthesiology, Stony Brook University, Stony Brook, NY, USA; 4 Department of Biochemistry, Stony Brook University, Stony Brook, NY, USA

**Keywords:** Reward, Ethanol, Fatty acid binding protein, Endocannabinoid, Addiction, Reward deficiency syndrome

## Abstract

The endocannabinoid (eCB) system is involved in a wide range of behavioral disorders including alcoholism. Inhibition of fatty acid amide hydrolase (FAAH), the principal enzyme that degrades the eCB anandamide (AEA), which enhances AEA levels in the brain, significantly increases ethanol consumption and preference. In the present study, we examined whether pharmacological inhibition of fatty acid binding proteins (FABPs) 5 and 7, which blocks the transport of AEA to FAAH, and increase AEA levels *in vivo* also alters ethanol consumption and preference. Using a limited access two-bottle choice paradigm, we evaluated ethanol consumption in both male and female C57Bl/6 mice. Results showed a significant decrease in ethanol consumption in both males and females treated with SBFI26, an inhibitor of FABPs. Specifically, male and female mice treated with SBFI26 consumed 24% and 42% less compared to mice receiving no injections, respectively. Subsequently, corticosterone was examined to evaluate the effects FABP5/7 inhibition upon the stress response. We observed a significant elevation in corticosterone levels following restraint stress in SBFI26 treated females, with a weak effect seen in males as compared to vehicle. Based on our results, targeting of FABPs appears to play an important role in ethanol consumption that is differentially regulated in males and females, which is mediated by the stress response.

## Introduction

There is a wealth of evidence linking the endocannabinoid (eCB) system with the physiological, behavioral, and rewarding effects of ethanol [1] and many other abused drugs [2]. eCBs are lipid retrograde messengers that activate cannabinoid type 1 receptors (CB1Rs), ubiquitously expressed in the brain, including brain areas involved in the regulation of reward [3]. Within the brain reward circuit, activation of CB1Rs regulates the strength and plasticity of glutamate [4] and GABA synapses [5, 6]. Through the regulation of synaptic transmission, eCBs modulate dopamine content in the nucleus accumbens (NAc) in response to ethanol and most other drugs of abuse [6, 7]. Blockade or deletion of CB1Rs reduces ethanol drinking behavior in mice [8, 9] with no associated characteristic increases in NAc dopamine release [10, 11]. In contrast, stimulation of CB1Rs has been shown to increase ethanol preference [12], indicating that an increase in eCB signaling facilitates ethanol drinking behaviors; although activation of CB1R in at least one structure of the brain has been shown to reduce ethanol consumption [13].

*N*-arachidonoylethanolamine (or anandamide/AEA) has been the most thoroughly studied eCB, particularly in ethanol research. Results from numerous studies have shown that inhibition of fatty acid amide hydrolase (FAAH) the primary enzyme that degrades AEA, results in a profound CB1R-dependent influence on the rewarding effects of ethanol in rodents. For instance, in an unrestricted two-bottle choice paradigm, FAAH−/− mice exhibit higher voluntary consumption of and preference for 12-20% ethanol without differences in sucrose/saccharine or quinine intake [14]. This effect was mirrored in mice treated with the FAAH inhibitor URB597 and blocked by CB1R antagonists [15]. One such study observed these increases only in female FAAH−/− mice [16], indicating that the sexual dimorphic effects of the eCB system may contribute to some of the sex-specific effects of ethanol observed in both preclinical and clinical studies [17]. While female wild-type mice typically consume more ethanol than males [18], no sex differences are observed in CB1−/− mice [10].

Emerging evidence has identified fatty acid binding proteins (FABPs) as intracellular chaperones of AEA. FABP5 is distributed at low levels throughout the whole brain and mainly found in neurons and glia cells, while FABP7 is mainly in glia cells localized at moderate levels in the olfactory bulb, dentate gyrus, and cerebellum and low levels throughout the rest of the brain [19]. Their inhibition or deletion blocks the transport of AEA from the plasma membrane to FAAH [20], thereby significantly increasing its availability in tissues where these FABPs are present [21–23]. Given that FABPs 5 and 7 are predominant in the brain [23], SBFI26 (a FABP5/7 inhibitor) may be advantageous over FAAH inhibitors like URB597 due to the fact that AEA elevation is more localized to the brain. Mice treated with SBFI26 and FABP5/7−/− mice display CB1R-dependent analgesic and anti-inflammatory reactions to a range of insults [21–23]. However, it is presently unknown whether inhibition of FABP5 and 7 influence the intake of commonly abused drugs such as ethanol.

A major caveat of compounds that manipulate the eCB pathways is the complexity of these pathways themselves (for a review, see Nicolussi and Gertsch [24]). AEA degradation is not exclusive to FAAH [5], and FAAH is also known to degrade other fatty acid amides including the NAEs *N*-palmitoylethanolamine (PEA) and *N*-oleoylethanolamine (OEA). FABP5 has been shown to carry these two NAEs to the nucleus where they bind peroxisome proliferator-activated receptor alpha (PPARα) [25] and regulate gene transcription [26]. All of these ligands and receptors have been shown to be implicated in the control of ethanol consumption [26–28]; however, the precise mechanism underlying these effects remains elusive.

Endocannabinoid signaling is also heavily involved in regulating stress, via the hypothalamic-pituitary-adrenal (HPA) axis. The tonic activation of CB1 by AEA helps restrain the activation of HPA axis through inhibitory neurons projecting to the paraventricular nucleus of the hypothalamus (PVN) [29, 30]. Previous studies have shown that elevated levels of AEA, due to inhibition of FAAH, can dampen the effects of stressors, thereby reducing corticosterone release [31]. Conversely, administration of CB1 antagonist prior to restraint stress exposure potentiated the release of corticosterone in mice [32]. Stressors have a large impact on voluntary ethanol consumption. Previous studies show decreases in ethanol consumption in C57BL/6J mice when exposed to restraint, tail suspension, foot shock, and tail pinch stress [33].

The present study examined whether FABP5 and FABP7 are involved in mediating ethanol consumption behavior. We hypothesized that mice treated with the FABP5/7 inhibitor would show increases in ethanol consumption, similar to previous data in mice treated with FAAH inhibitors. Lastly, given the increases in brain levels of AEA due to SBFI26, we examined how SBFI26 affects corticosterone release and the role it may play in regulating ethanol intake.

## Methodology

### Subjects

9 week old C57Bl/6 mice (n = 48, 25 g for males, n = 48, 20 g for females) were purchased from Taconic (Hudson, NY) and singled housed in a temperature (22 °C) and humidity controlled room on a reverse 12 hour light cycle (9:00-21:00). All mice were handled and habituated to their new environment for a period of 1 week. Food and water were provided *ad libidum*. Body weight and food intake were also monitored daily. All mice began testing at 10 weeks of age. All procedures followed the National Institutes of Health Guidelines for the Care and Use of Laboratory Animals and were approved by the University at Buffalo Institutional Animal Care and Use Committee.

### Drug

The FABP inhibitor SBFI26 was synthesized as in [21]. The SBFI26 drug was dissolved in a 4% DMSO, 10% Kolliphor EL, 86% saline solution and made daily, prior to administration. SBFI26 was administered at a dose of 20 mg/kg, as this dose has been previously demonstrated to significantly elevate brain levels of AEA in mice [23].The vehicle group received the same vehicle solution without the drug. Injections were administered intraperitoneally daily, at a volume of 10 μl/g body weight, immediately before the mice received bottles. Ethanol bottles were prepared by diluting 200 proof ethanol (Pharmaco-AAPER, Brookfield, CT) with distilled water to the desired concentration of either 3%, 6%, or 12% (v/v).

### Drinking in the dark paradigm

The drinking in the dark procedure, implemented by [34], was used for this experiment. Mice were given access to two Falcon tube bottles, one with water and the other with ethanol. The first three trials were for the purpose of habituating the mice to this paradigm, starting at a concentration of 3% ethanol for three days, followed by 6% for another three days, continuing to 12% ethanol. Trial 3 was used as the baseline consumption for ethanol at 12%. During trials 4–6, the mice were still given 12% ethanol but they were either given an injection of the vehicle or SBFI26 or they were not given an injection at all. Bottles were given one hour into their dark cycle, left for 6 hours, and weighed immediately afterwards. A single water bottles was returned immediately following the removal of the bottles. Ethanol and water intake, along with food and body weights, were recorded daily. All bottles were washed and refilled in between each trail. A dummy bottle for both ethanol and water was placed in an empty cage to account for spillage or evaporation. The amount consumed was calculated by subtracting the amount lost from the dummy from the total amount consumed.

### Corticosterone analysis

Prior to sacrifice, mice received their respective injections and were either physically restrained or remained in their home cage. Injections were performed 2 hours prior to being anesthetized. This was done so that the SBFI26 drug would produce peak raises in anandamide at 90 minutes [23] which is during the middle of the restrained period). The stress group was physically restrained in a Falcon tube for 30 minutes to produce peak corticosterone levels [35]. The tubes contained multiple holes, providing ventilation for the mice. Following anesthesia with isoflurane, blood was removed via cardiac puncture and allowed to clot for 30-45 minutes before being spun at 800 × g at 4 °C for 15 minutes. Serum was stored at −80 °C until analysis with an IBL Mouse/Rat Corticosteroid ELISA Kit (REF IB79175).

### Statistical analysis

Ethanol dosages were calculated based on total grams of ethanol consumed over daily body weights. Grams of ethanol consumed was calculated based off the percent ethanol in solution and the volume of solution consumed. Preference for ethanol was evaluated by dividing the volume of ethanol solution consumed by the total combined fluid volume consumed per subject per day. Three-day means of the (+) SBFI26 trial were compared to baseline (trial 4-6) means. The Grubbs tests was performed to identify outliers prior to running statistics. Statistical significance was set at α = 0.05. All statistics and graphing were conducted using SigmaPlot 11.0 (Systat Software Inc., San Jose, CA).

## Results

### Consumption

**Figure 1:**
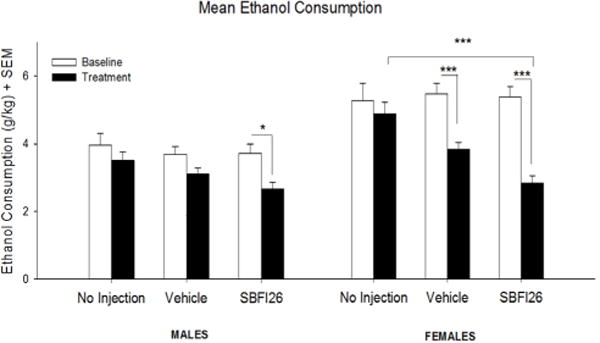
Mean (+SEM) ethanol consumption in a two-bottle choice ethanol paradigm in both male and female mice. *p < 0.05; ^***^p < 0.001.

A three-way repeated measures ANOVA was conducted to examine the effects of Sex [Male, Female], Treatment [Control, Vehicle, SBFI26] over the course of Trial [Baseline, Test] on voluntary ethanol consumption (Figure 1). Significant effects were observed for Sex [*F*(1,88) = 34.609, *p* < 0.001], Treatment [*F*(2,88) = 4.613,*p* < 0.05], Trial [*F*(1,88) = 77.02, *p* < 0.001], Trial x Sex [*F*(1,88) = 12.714,*p* < 0.001] and Trial X Treatment [*F*(1,88) = 10.042, *p* < 0.001]. Post-hoc tests using Tukey’s HSD showed females to consume 35% (*p* < 0.001) more than males, overall. The male SBFI26 treated mice consumed 29% less during the test trial compared to baseline (*p* < 0.05). The female vehicle group consumed 30% less during the test trial compared to baseline (*p* < 0.001). Similarly, the female SBFI26 group consumed 47% less during the test trial compared to baseline (*p* < 0.001). During the test trial, the female SBFI26 treated mice consumed 41% less than the control treated mice (*p* < 0.001). No significant difference was seen between the control and vehicle group during the test trial.

A three-way repeated measures ANOVA was also conducted to examine the effects of Sex [Male, Female], Treatment [Control, Vehicle, SBFI26] over the course of Trial [Baseline, Test] on food consumption, water consumption, and total fluid consumption. A main effect was seen in food consumption (data not shown) for Sex [F(1,89) = 80.985, p < 0.001], where females consumed more food than males.

A main effect was seen in water consumption (data not shown) for Sex × Group [F(2,90) = 10.58, p < 0.001] and Trial [F(1,90) = 20.1532, p < 0.001]. Water consumption increased between during the testing period compared to baseline. Post-hoc tests using Tukey’s HSD revealed control females consumed more water compared to control males (p < 0.001). Control females also consumed more water during the test period than the female vehicle group (p < 0.001) as well as the female SBFI26 group (p < 0.001).

Main effects in total fluid consumption (data not shown) were found for Sex [F(1,90) = 31.458, p < 0.001], Treatment [F(1,90) = 7.432, p < 0.01], Sex x treatment [F(2,90) = 14.678, p < 0.001], Trial [F(1,90) = 9.108, p < 0.05], Trial x Sex F(1,90) = 5.454, p < 0.05], Trial x treatment [F(2,90) = 5.931, p < 0.05], and Trial x Sex x treatment [F(2,90) = 8.042, p < 0.001]. Control female consumption during test was greater compared to the female vehicle group (p < 0.001) and the female SBFI26 group (p < 0.001). The female SBFI26 group consumed more during the test period compared to baseline, while the control and vehicle groups did not show this difference between phases (*p* < 0.001). Moreover, control females consumed more during baseline than control males; the same was also true during the test period (*p* < 0.001).

### Ethanol preference

A three-way repeated measures ANOVA was also used with the same factors above to examine changes in preference for the (12%) ethanol bottle. Significant main effects were found for Sex [F(1,88) = 39.661, *p* < 0.001] and Sex x Treatment [F(2,88) = 9.804,*p* < 0.001]. In the male SBFI26 treated mice, preference for ethanol decreased by 31% during the treatment compared to baseline preference (*p* < 0.001). No other groups showed changes in preference.

### Corticosterone

**Figure 2:**
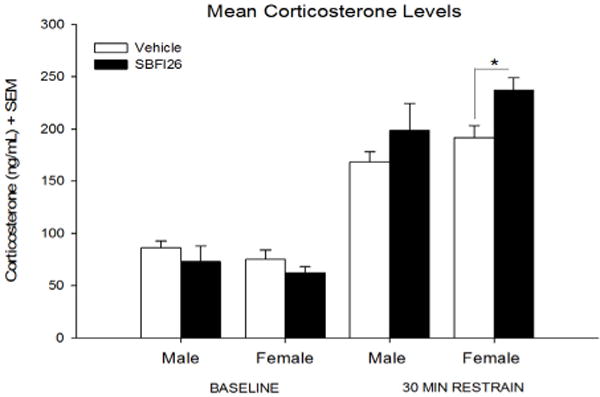
Mean (+SEM) plasma corticosterone levels in male and female mice at baseline and following a 30 minute restrain. *p < 0.05.

Two separate two-way ANOVAs were used to assess the effects of Sex [Male, Female], Treatment [Vehicle, SBFI26] under basal conditions and after 30 minutes of physical restraint on corticosterone levels. Significant effects were observed in Treatment [F(1,26) = 5.99, p < 0.05; Figure 2] within physical restraint conditions. Post-hoc comparisons revealed a group difference in female (p < 0.05), while males had a weaker, non-significant effect (p = 0.17). No significant difference was seen under basal conditions for Sex [Male, Female] nor among Treatment [Vehicle, SBFI26].

## Discussion

Over the past few decades considerable attention has been given to the role of the eCB system in controlling drug intake and addiction, including ethanol addiction. Others have demonstrated that FAAH inhibition as well as FAAH gene deletion leads to significant increases in ethanol preference and consumption [14, 15]. These behavioral effects have been attributed to AEA-mediated activation of a CB1Rs. Dopaminergic projections from the ventral tegmental area (VTA) to the NAc play a critical role in reward processing, with increases in NAc dopamine levels leading to positive reinforcement. CB1R agonists, like AEA, increase DA levels in NAc in a CB1R-dependent manner, thereby increasing the reinforcing effects of ethanol. Whereas obstruction of CB1Rs in the VTA, as well as NAc, decreases ethanol self-administration and ethanol consumption (for a review see [36]). Accordingly, inhibition of FABP5 and 7, which enhances AEA levels should increase ethanol consumption and preference. Surprisingly, in the current study, we find that unlike FAAH inhibition, the inhibition of FABP5 and 7 decreased both the preference and consumption of ethanol in mice, indicating either an unknown mechanism of the SBFI26 drug or FABPs. These results suggest that the inhibition of FABP5 and 7 may be hindering AEA ability to interact with CB1Rs, despite the elevated levels of the neurotransmitter.

Preference for ethanol in males was significantly decreased by 31% after receiving the SBFI26 drug, whereas preference in females decreased by 18%. Ethanol consumption decreased in both males and females. Compared to a baseline, male mice receiving SBFI26 showed a 26% decrease in ethanol consumption. Within the SBFI26 treated mice, females showed a 47% decrease in ethanol consumption during the treatment. While females typically consume more ethanol than males under normal conditions, it seems that the impact of repeated daily injections diminished female consumption to the point where it was similar to the males. The female vehicle group showed a reduction in their ethanol intake by 22% compared to the control group, which was not significant. However, females receiving the SBFI26 drug showed a significant 42% decrease in consumption when compared to the control. As previously mentioned, CB1Rs are important to the sex- dependent differences seen between males and females, with respects to ethanol consumption. If the inhibition of FABP5 and 7 is hindering AEA ability to interact with CB1Rs, then sex-dependent differences would be eliminated, as seen in CB1−/− male and female mice [10]. However, it remains unclear why the decrease in consumption due to vehicle alone was able to eliminate sex differences. Since the control groups were not restrained daily and given an injection, it is possible that this decrease in the vehicle group may be due to the impact of stress. Previous studies have demonstrated that restraint stress significantly decreased ethanol consumption without producing a sex-dependent effect [33].

Under basal physiologically conditions, AEA signalling within the basolateral amygdala (BLA) inhibits glutamatergic transmission and the activation of the PVN through transsynaptic pathways within the amygdala [30]. This will, at least in part, reduce the initiation of the stress response. Exposure to stress increases the FAAH activity, thereby reducing levels of AEA and enhancing glutamatergic transmission onto BLA neurons, which mediate stress responses [29]. Corticosterone levels were examined in non-stressed and stressed animals between treatment with either a vehicle or SBFI26 injection. We expected that since SBFI26 have been shown to increase AEA levels, a decrease in stress-induced corticosterone would be seen. Unexpectedly, females injected with SBFI26 showed a 24% increase in corticosterone levels following restrain stress. A weak effect was seen in males receiving SBFI26, exhibiting an 18% increase in corticosterone. While this difference is not significant, further investigation may be required. Taken together, these results support the claim that the decreases in ethanol consumption may have been stress related. Palmer and colleagues have demonstrated that mice overexpressing CRF, which is released due to the activation of the PVN [30], showed decreased ethanol consumption and increased ethanol sensitivity [37]. While the exact mechanism is unclear, the increased corticosterone response could explain the decrease in consumption of ethanol.

FABPs have also been reported to transport endocannabinoids to other targets such as the vanilloid receptor 1 (TRPV1) [22]. AEA is a ligand for the TRPV1 receptor. Deletion of TRPV1 has been shown to increase ethanol consumption [27]. If TRPV1 activation has an opposing role to CB1Rs, as purposed by Blednov and colleagues, the elevated AEA could have caused the decrease in consumption via a TRPV1-dependent mechanism. Recently, Peng and colleagues examined the mechanism underlying the analgesic properties of SBFI26. It was reported that no CB1-mediated effects were observed, despite the elevated AEA levels reported after intracerebroventricular administration of the inhibitor [38]. It was suggested that inhibition of FABPs may interfere with the delivery of endocannabinoids to CB1Rs. Others have speculated that eCB transporters like FABPs are necessary to transport AEA to the cell membrane to be released from the cell either through facilitated diffusion across the plasma membrane or mediated by a putative eCB membrane transporter (see review Nicolussi and Gertsch [24]).

Interrupting the delivery of AEA to CB1Rs, could explain why the elevated AEA did not facilitate the CB1R-mediated increase in ethanol consumption similar to what is observed when FAAH is inhibited or knockout. As previously stated, interaction with CB1Rs in the BLA is crucial to the feedback regulation of the HPA axis. Studies have shown that antagonism of the CB1 receptor leads to elevated corticosterone and delays the negative feedback response of the HPA axis [32, 39–41]. If FABPs mediate the transport of AEA to membrane CB1 receptors, inhibition of FABPs may have a similar effect to what others have found showing increased corticosterone levels as a result of CB1 antagonism in the BLA. Moreover, the decreased activation of CB1Rs would result in decreases in NAc dopamine levels consequently preventing the reinforcing effects of ethanol. Nevertheless, the biological and behavioral effects of SBFI26 are still not well known and therefore it is possible that these results are due a side effect not yet discovered.

## Conclusion

The eCB system is clearly tied in the regulation of reward- related behavior, ethanol consumption and potentially in reward deficiency syndrome [42]; however, relatively little is known about these interactions (for a review, see [5, 43]. The current study has demonstrated that the administration of FABP5/7 inhibitor, SBFI26, decreased consumption in both males and females by 24% (*p* < 0.05) and 42% (*p* < 0.001) less when compared to the group receiving no injections. The reduction in consumption seen in the vehicle group indicates that that the reduction in ethanol consumption may be stress mediated. Corticosterone analysis shows that SBFI26 elevated corticosterone by 18% in males and 24% females, suggesting that FABP 5/7 inhibition increases sensitivity to stressors. Given its relatively recent creation and synthesis, there is still a great deal that is unknown with reference to both the biological and behavioral effects of SBFI26. Although SBFI26 has been demonstrated to possess anti-inflammatory and pain relieving effects [23] the degree to which this drug can alter reward related behavior remains to be fully elucidated.

## Abbreviations

AEA: Anandamide
BLA: Basolateral Amygdala
CB1Rs: Cannabinoid Type 1 Receptors
eCB: Endocannabinoid
FAAH: Fatty Acid Amide Hydrolase
FABP: Fatty Acid Binding Protein
HPA: Hypothalamic-Pituitary- Adrenal Axis
NAc: Nucleus Accumbens
PPARα: Peroxisome Proliferator- Activated Receptor Alpha
PVN: Paraventricular Nucleus of Hypothalamus
VTA: Ventral Tegmental Area
